# Flexible Superconducting Wiring for Integration with Low-Temperature Detector and Readout Fabrication

**DOI:** 10.1007/s10909-024-03209-8

**Published:** 2024

**Authors:** Galen O’Neil, Daniel Swetz, Randy Doriese, Dan Schmidt, Leila Vale, Joel Weber, Robinjeet Singh, Mark Keller, Michael Vissers, Kelsey Morgan, John Mates, Avirup Roy, Joel Ullom

**Affiliations:** 1 National Institute of Standards and Technology, Boulder, CO, USA; 2 University of Colorado, Boulder, Boulder, CO, USA

**Keywords:** Cryogenic, Silicon, Niobium, Aluminum, Molybdenum, Critical current, Superconductor

## Abstract

We present a method of creating high-density superconducting flexible wiring on flexible thin silicon substrates. The flexible wiring, called *SOI flex*, is created by depositing superconducting wiring on a silicon-on-insulator (SOI) wafer, selectively etching away the thicker silicon section *handle* layer, and bending the thinner silicon *device* layer. We show measurements of superconducting transition temperature and critical current for Mo, Nb, and Al on SOI flex. We discuss the expected advantages of SOI flex for low-temperature detector applications, as well as the role of stress and strain in bent silicon and niobium.

## 1 Introduction

Flexible circuits are often used at room temperature as connectors in applications where flexibility or space savings are critical. The same properties are desirable for low-temperature detector (LTD) applications. LTD applications often additionally require superconducting or at least very low resistance wiring. Many flexible wiring solutions exist commercially, but most do not support superconducting materials necessary for LTDs. Here, we present *SOI flex*, which provides a flexible circuitry solution that easily integrates into existing LTD detector and readout fabrication processes, works with many superconducting materials, supports high wiring density, and can serve as a weak thermal link when desired. We expect SOI flex to be compatible with many microwave frequency applications, based upon simulations of the expected loss. The use of flexible silicon within LTD applications goes back at least to the SHARC-II camera [1].

Some LTD applications such as soft X-ray spectrometers, based on large arrays of transition edge sensors (TES), benefit from an out-of-plane design. In these out-of-plane designs, the readout circuity, which is often larger on a per-pixel basis than the sensors themselves, is located on a surface perpendicular to the plane of the sensors. Out-of-plane designs require less area in-plane, which allows the surrounding magnetic shields, radiation shields, and vacuum components to be more compact than with an in-plane design. Flexible superconducting circuitry is used to connect the in-plane sensors to the out-of-plane readout circuits. So far, wirebonds have been used to connect from the sensors to the flexible circuits and from the flexible circuits to the sensors. Alternatively, the wirebonds have been used as the flexible circuits themselves [2]. In these cases, the wiring density is limited by the density of wirebonds for which 200 um pair pitch is routine; and 50 um is plausible with bond-over-bond methods. In comparison, SOI flex is compatible with a wide range of optical lithography and thin-film deposition techniques, so a 10 um pair pitch with microstrip wiring running over SOI flex should be easily achievable.

Consider a possible out-of-plane X-ray TES array with 1000 mm^2^ active area, with the goal of requiring minimum in-plane area. A 10,000 pixel array on a 330 um pitch is plausible based on pixel designs for the ATHENA X-IFU [3]. For a square array, the side will be 31.6 mm, and the available perimeter for around-the-corner wiring is 126 mm. The signal wire pairs must be on a 12.6 um or tighter pitch. This pitch is not achievable with previously demonstrated superconducting around-the-corner wiring solutions. Microstrip wiring on SOI flex with 10 um pair pitch appears to be a viable path to build such an array. The readout circuitry would either be fabricated on the same wafer or connected by wirebonds distributed throughout the area of the four out-of-plane surfaces.

## 2 Methods

We fabricated test structures consisting of two full-thickness (400 um) wafer sections, connected by a flexible section of 9.4 mm by 1.7 mm and thickness 2 um. Traces of Cu, Mo, Nb, and Al extended from one side to the other and back, such that electrical transport properties of wires on the flexible section can be interrogated with wirebonds on only one side of the test structure. We began with a commercial SOI wafer consisting of a thin 2 um *device* layer, an 0.2 um buried oxide layer, and a thick 400 um *handle* layer. We used established processes for depositing metal films on the device side Si, they were not altered for this work. The Al layer was deposited with electron beam deposition and patterned with a bilayer resist lift-off mask, with a deposition rate 0.5 nm/sec. The Mo layer was deposited with DC-magnetron sputtering and patterned with a phosphoric acid-based wet etch using a photo-resist mask, with a deposition rate of 0.5 nm/s. The Nb layer was deposited with DC-magnetron sputtering and patterned with a bilayer resist lift-off mask, with a deposition rate of 0.4 nm/s. The pressure during Mo and Nb sputtering was previously tuned to yield films with slightly compressive stress [4]. After patterning, the device layer is etched in an inductively coupled plasma etch chamber using a Bosch-type process. With the same patterning, the buried oxide layer is etched with a parallel plate reactive ion etch system using a O2:CHF3 gas mixture. Then, backside processing begins, first the device side of the wafer was mounted to a sapphire carrier wafer using a clear wax. After patterning, the handle layer is etched with a deep reactive ion etcher with a Bosch process to achieve vertical sidewalls. We did not remove the buried oxide layer on the backside. Finally, we released the structure by dissolving the wax in successive acetone baths. The structures were picked up with tweezers, taking care to handle only the larger silicon block and bringing the device through the solvent–air interface as perpendicular as possible.

**Figure. 1:**
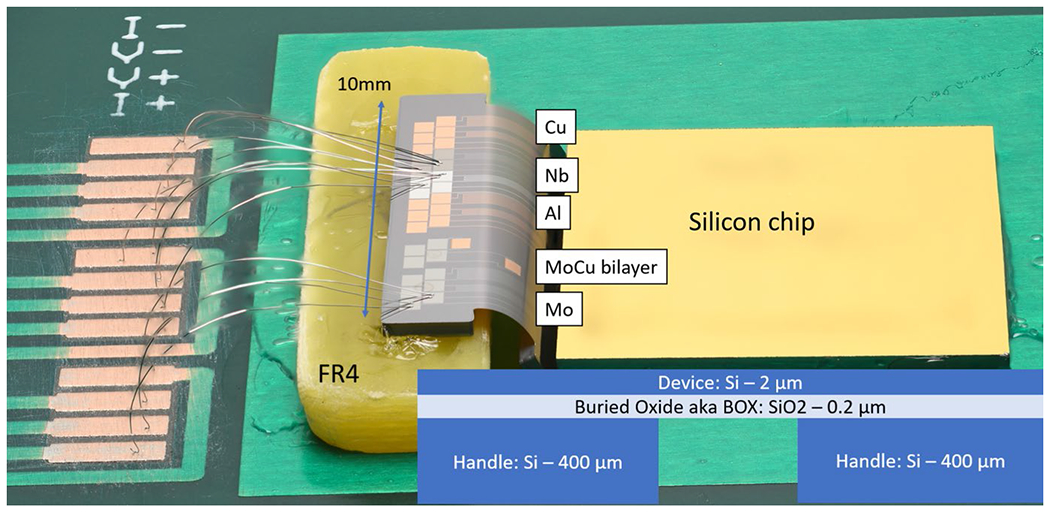
SOI flex test structure. Consists of Si sections with dimensions 10 mm by 2 mm and 10 mm by 1.8 mm connected by one flexible section with dimensions 9.4 mm by 1.7 mm. The SOI flex is attached with rubber cement to a hand-cut section of FR4 and held in a bent configuration by a nearby Si chip. Metal test structures with bond pads are visible and labeled with their composition, wirebonds are visible with artifacts from photography using focus combination. Inset in the lower right is a cross-section of the Si layers of the device. The bend radius *r* = *d/θ* where *d* =1.7 mm is the length of the SOI flex, and *θ* = 65 degrees is estimated from this photo. We arrive at a bend radius of 1.5 mm.

A test structure was mounted to a block of FR4 and bent by force from a Si chip placed nearby. All items were attached with rubber cement. This test structure is shown in Fig. 1 and has a bend angle of 65 degrees. We cooled this structure down in an adiabatic demagnetization refrigerator. We measured the resistance as a function of temperature to determine the superconducting transition temperature, and we used a pulsed current method to measure critical current with minimal heating. The results, in Table 1, are consistent with regularly achieved values for these materials and sufficient for the LTD applications we currently envision.

## 3 Microwave Loss

**Figure. 2:**
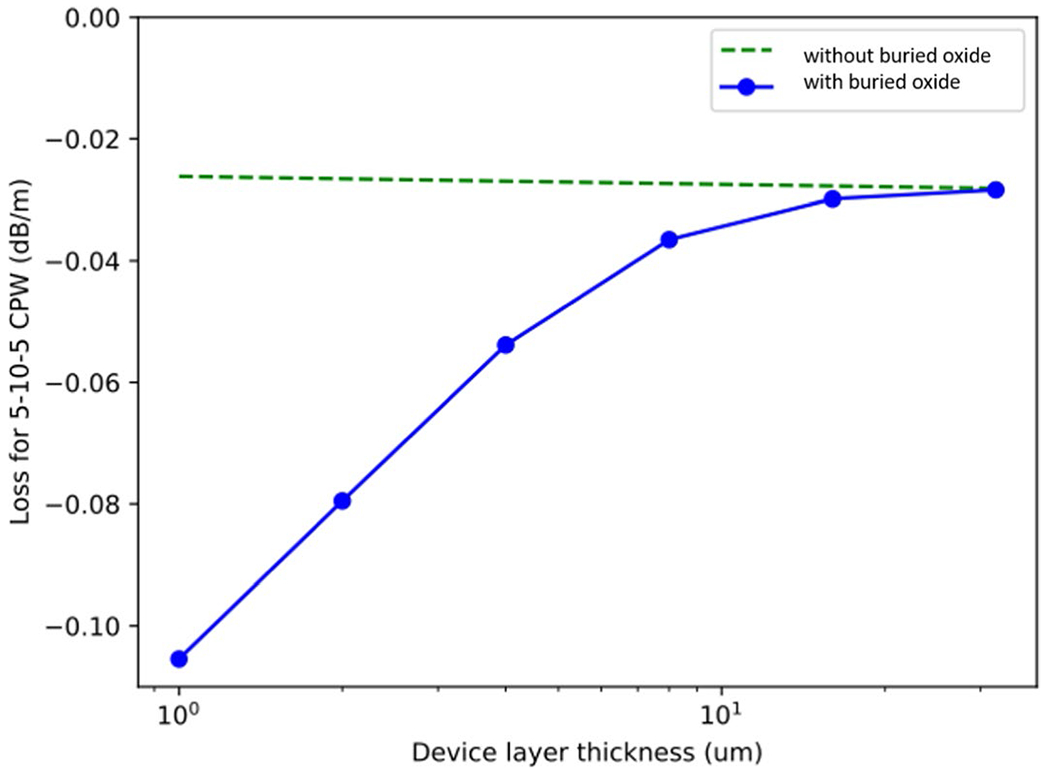
Loss per meter in a superconducting co-planar waveguide on either 25 nm surface oxide (green) or 25 nm surface oxide plus 0.2 um buried oxide below the device layer. The simulations were done in Sonnet, and the co-planar waveguide had a 10 um center conductor with 5 um gaps. For a device layer thickness of 4 um, the loss with the buried oxide is about twice the loss with only the surface oxide.

Many LTD applications use superconducting microwave resonators and transmission lines with signals in the 4–8 GHz range. Oxides near these structures, such as the buried oxide layer in the SOI wafer, add loss and two-level system noise. We envision fabricating microwave squid multiplexers on SOI wafers. We aim to keep the loss due to buried oxide to equal or less than the loss of the 25 nm oxide layer regularly used with microwave squid multiplexers [5]. We simulated the loss in a co-planar waveguide on Si wafer with a 25-nm-thick surface oxide layer and a 0.2-um-thick buried layer oxide layer below the device layer. Figure 2 shows the loss calculated for this co-planar waveguide as a function of device layer thickness, as compared to the loss with no buried oxide. For a 4-um-thick device layer, the loss with buried oxide is twice the loss with no buried oxide. So, the contribution to loss from the buried oxide alone is comparable to that of a surface oxide. Therefore, we expect that SOI wafers with micron scale devices layers are likely to be compatible with most superconducting microwave applications, especially those that already rely on a surface oxide layer.

## 4 Stress

Bending a thin layer strains the material, which may impact the superconducting properties of wiring and will cause breakage of the Si device layer or wiring. We envision fabrication processes with 4-um-thick device layer and bend radius of 0.5 mm or greater. Here, we estimate the limit on bend radius imposed by the breaking strain of Si. A bend is characterized by the bend radius R, assuming the bent surface lies along a circle. The outer and inner surfaces of the bend will have strain of magnitude *ϵ* = *t*/2*R* and opposite sign, where *t* is the thickness. The mechanical stress is then *σ_st_* = *Eϵ* where *E* is the Young’s modulus. Si forms an anisotropic crystal, so the Young’s modulus depends on the orientation and loading of the structure, the value *E* = 169 GPa is recommended for in-plane stresses of a (100) wafer as used here [6]. For the current structure with *t* = 2 um and *R* = 1.5 mm, the strain is 7×10^−4^, and the stress is 0.11 GPa. The breaking stress of Si depends on the presence of microcracks, the highest reported values approach 18 GPa. A study using fabrication techniques similar to those used here reports the minimum breaking stress across 20 devices was 2 GPa [7]. For a device layer thickness of 4 um, a maximum allowed stress of 1.5 GPa would permit a bend radius as small as 0.26 mm.

Here, we consider the effect of strain on superconducting wiring running over SOI flex. If the wiring is thin compared to the SOI flex, the strain in the wiring should match the peak strain in the Si. The transition temperature in superconducting films depends on strain; a recent calculation predicts transition temperatures above 6 K for strain as large as 0.04 in niobium [8]. Materials elastically deform below their yield stress and plastically deform beyond the yield stress. If we take a reasonable value of 50 MPa for the yield stress in niobium, then we find that niobium wiring begins to plastically deform for bend radiuses below 4 mm with a device layer thickness of 4 um. The breaking strain in niobium is larger than 0.4 [9, 10]. Imamura et al. reported superconductivity in niobium films with both compressive and tensile stress up to 400 MPa [4]. The breaking strain in both aluminum and molybdenum can be over 0.1 [11, 12]. The transition temperature of aluminum increases with strain up to at least 0.04 [13]. The strain for an envisioned 0.5 mm bend radius structure on 4-um-thick device layer is 5×10^−3^, which is much smaller than the critical strain values discussed here. It is unlikely that aluminum, molybdenum, or niobium properties will limit the minimum bend radius more than Si properties.

## 5 Thermal Conductivity

We estimate the phonon contribution to thermal conductivity *G* of SOI flex with this expression based upon the kinetic theory of gasses

$$G=\gamma T^{3}\frac{\mathrm{cl}}{3}\frac{A}{L}$$

where $\gamma =0.57\frac{1}{{\text{K}}^{4}{\text{m}}^{3}},\;\gamma T^{3}$ is the phonon contribution to the heat capacity in Si [14], *T* is the temperature, $l=\sqrt{2}t$ is an estimate of the phonon mean free path assuming it is limited by the thickness, *A* is the cross-sectional area, and *L* is the length. The speed of sound in Si $c=\sqrt{E/\rho }=8520\frac{\text{m}}{\text{s}}$ where *ρ* = 2.329 g/cm^3^ is the density of Si. Si features have been used in many LTD applications to set thermal conductivity of devices, including the aforementioned SHARC-II bolometers [1]. For soft X-ray TESs with typical dimensions on order 100s of um, transition temperature of 50 mK, 30 pW/K is a representative thermal conductivity. Based on the above expression, a SOI membrane with width 130 um, length 20 um, and thickness 4 um would have a thermal conductivity of 30 pW at 50 mK. A silicon feature of these dimensions would be easily incorporated into a soft X-ray TESs to set the thermal conductivity.

Most, if not all, LTD applications require routing electrical signals among different temperature stages of cryostats. At the lowest temperature stages, with the least cooling power available, NbTi twisted pairs or NbTi coaxial cables are widely used depending on the bandwidth requirements. Much effort associated with LTD applications is devoted to multiplexing techniques, which reduce the wire counts necessary between temperature stages to reduce thermal loads, space requirements, and cost. The small dimensions and high wiring densities achievable with SOI flex present an opportunity to run many wires among temperatures stages, with no connectors necessary if devices are fabricated on the same chip. For example, one could fabricate LTDs intended for base temperature stage on one portion of a chip and power-hungry amplifiers intended for a higher temperature stage on another portion of the same chip, and connect them with wiring running on SOI flex. Such a device would be challenging to mount in a cryostat, but the thermal and electrical properties are quite favorable. As a point of comparison, a 30 cm long section of 0.86 mm outer diameter NbTi coax has a thermal conductivity of 154 nW/K at 4 K [15]. A section of SOI flex with width 0.7 mm, thickness 4 um, and length 10 mm is estimated to contribute 163 nW/K at 4 K, which would be increased somewhat by the addition of superconducting wiring to carry the signals. The thermal conductivity of both the coax and the SOI flex is dominated by phonons, so the temperature dependence should be near *T*^3^ in both cases. For the case of microstrip wiring with 10 um pitch, the 10 mm long SOI flex could carry 70 microwave signals with roughly the same thermal load as one NbTi coaxial cable while using much less space than 70 coaxial cables.

## 6 Comparison to Polyimide Flex

Flexible superconducting wiring based on polyimide has been developed. Fabrication of superconducting wiring on polyimide flex requires 1) modified etching to create sloped sidewalls due to the relatively large thickness; 6.5 um in one case, 2) baking at 350 C that may modify critical LTD properties such transition temperatures or junction resistances, and 3) seed layers to promote niobium adhesion and achieve good transition temperatures [16–18]. These additional fabrication requirements make polyimide flex difficult to integrate with active device fabrication. So, for applications where devices will be fabricated on the same wafer and wired together, SOI flex has significant advantages. Polyimide flex has advantages for larger scale wiring between separate chips or devices that need to be handled as it is much more robust.

## 7 Conclusions

SOI flex is a promising tool for the development of large arrays of LTDs that have space or wiring density requirements that cannot be easily met with wire bonds. SOI flex is easy to integrate into many LTD fabrication processes, including those that use microwave frequencies. The same fabrication process that creates SOI flex can make thin Si structures to limit thermal conductivity where desired.

## Figures and Tables

**Table 1:** Transition temperature, critical current, and resistance per square measured at 300 K and 3 K, for 10 um wide Al, Mo, and Nb

Metal	Substrate	Tc (K)	Ic (mA)	Rsq 300 K (Ω/sq)	Rsq 3 K (Ω/sq)
Al	Solid	1.2	13	0.30	0.05
Al	Flex free	1.2	3.9	0.30	0.05
Al	Flex bent	1.2	12	0.30	0.05
Al	Flex bent	1.2	4.5	0.30	0.05
Mo	Solid	1.1	3.1	2.96	1.87
Mo	Flex free	1.1	2.9	2.94	1.84
Mo	Flex bent	1.1	2.9	3.00	1.89
Nb	Solid	8.0	74	0.46	0.00
Nb	Flex free	8.0	76	0.46	0.00
Nb	Flex bent	8.0	74	0.46	0.00
Nb	Flex bent	8.0	76	0.46	0.00

The films thicknesses were 100 nm for Al, 60 nm for Mo, and 400 nm for Nb. Samples with the same patterning except for the backside deep-etch were measured on a solid substrates, “flex bent” substrates which were bent as shown in Fig. 1, and “flex free” substrates which had relieved SOI flex with one end glued down and the other end free floating so that the SOI flex is mostly flat. Critical currents were measured at 85 mK for Mo and Al, and 3K for Nb. Al traces had a large spread in critical currents from 4 to 13 mA. Because one “flex bent” Al trace had 12 mA critical current, we suspect that the spread is unrelated to the flexible substrate. The measured properties of the superconducting films on flexible substrates are sufficient for transition edge sensor applications and do not show significant differences from those on solid substrates

## References

[1] Voellmer GM , “Design and fabrication of two-dimensional semiconducting bolometer arrays for HAWC and SHARC-II,” Millimeter and Submillimeter Detectors for Astronomy, vol. 4855, no. February, p. 63, 2003, 10.1117/12.459315.

[2] Szypryt P , A Tabletop X-Ray Tomography Instrument for Nanometer-Scale Imaging: Demonstration of the 1,000-Element Transition-Edge Sensor Subarray. IEEE Trans. Appl. Supercond 33(5), 1–5 (2023). 10.1109/TASC.2023.3256343

[3] Miniussi AR , Performance of an X-ray Microcalorimeter with a 240 μm Absorber and a 50 μm TES Bilayer. J. Low Temp. Phys 193(3–4), 337–343 (2018). 10.1007/s10909-018-1974-4

[4] Imamura T, Shiota T, Hasuo S, Fabrication of high quality Nb/AlO/sub x/-Al/Nb Josephson junctions. I. Sputtered Nb films for junction electrodes. IEEE Transactions on Appiled Superconductivity 2(1), 1–14 (1992). 10.1109/77.124922

[5] Dober B , “A microwave SQUID multiplexer optimized for bolometric applications,” Appl Phys Lett, vol. 118, no. 6, 2021, 10.1063/5.0033416.

[6] Hopcroft M, What is the Young ‘ s Modulus of Silicon ? What is the Crystal Orientation in a Silicon Wafer ? Physical Acoustics 19(2), 229–238 (2007)

[7] Gaither MS, Delrio FW, Gates RS, Fuller ER, Cook RF, Strength distribution of single-crystal silicon theta-like specimens. Scr. Mater 63(4), 422–425 (2010). 10.1016/j.scriptamat.2010.04.047

[8] Choi J, Kim YK, Kim CD, Kim S, and Jo Y, “Enhancing the critical temperature of strained Niobium films,” Mater Res Express, vol. 7, no. 7, 2020, 10.1088/2053-1591/aba84a.

[9] Antoine C, Foley M, and Dhanaraj N, “PHYSICAL PROPERTIES OF NIOBIUM AND SPECIFICATIONS FOR FABRICATION OF SUPERCONDUCTING CAVITIES,” Fermilab Technical Division Note, no. TD-06-048, 2006, [Online]. Available: https://lss.fnal.gov/archive/test-tm/2000/fermilab-tm-2503-td.pdf

[10] Kostin R ,(2015) “A tuner for a superconducting traveling wave cavity prototype,” Journal of Instrumentation 10 10. 10.1088/1748-0221/10/10/P10038.

[11] Yun X, Wang Z, and Gardner L, “Full-Range Stress-Strain Curves for Aluminum Alloys,” Journal of Structural Engineering, vol. 147, no. 6, 2021, 10.1061/(asce)st.1943-541x.0002999.

[12] Wang C, Wan M, Zhou Y, and Wu X, “A ductile fracture criterion with Zener-Hollomon parameter of pure molybdenum sheet in thermal forming,” MATEC Web of Conferences, vol. 21, no. January, 2015, 10.1051/matecconf/20152107006.

[13] Notarys HA, Effect of tensile strain on superconducting transition temperature of Al films. Appl. Phys. Lett 4(4), 79–80 (1964). 10.1063/1.1753971

[14] Pearlman N, Keesom PH, The Atomic Heat of Silicon below 100°K. Phys. Rev 88(2), 398–405 (1952). 10.1103/PhysRev.88.398

[15] “http://www.coax.co.jp/en/product/sc/086-50-nbti-nbti.html.”

[16] Chervenak JA , Broadside-Coupled Niobium Flexible Cables. IEEE Trans. Appl. Supercond 33(5), 5–9 (2023). 10.1109/TASC.2023.3252479

[17] DeNigris NS , Fabrication of Flexible Superconducting Wiring with High Current-Carrying Capacity Indium Interconnects. J. Low Temp. Phys 193(5-6), 687–694 (2018). 10.1007/s10909-018-2019-8 31359888 PMC6662641

[18] Gupta V , Thin-film Nb/Polyimide superconducting stripline flexible cables. IEEE Trans. Appl. Supercond 29(5), 1–5 (2019). 10.1109/TASC.2019.2904203

